# Expanded Use of Vorasidenib in Non-Enhancing Recurrent CNS WHO Grade 3 Oligodendroglioma

**DOI:** 10.3390/biomedicines13010201

**Published:** 2025-01-15

**Authors:** Alexander S. Himstead, Jefferson W. Chen, Eleanor Chu, Mari A. Perez-Rosendahl, Michelle Zheng, Sherin Mathew, Carlen A. Yuen

**Affiliations:** 1 Department of Neurological Surgery, University of California, Irvine, CA 92697, USA; ahimstea@hs.uci.edu (A.S.H.); jeffewc1@hs.uc.edu (J.W.C.); 2 Department of Radiological Sciences, University of California, Irvine, CA 92697, USA; eleanoc@hs.uci.edu; 3 Department of Pathology & Laboratory Medicine, University of California, Irvine, CA 92697, USA; mperezro@hs.uci.edu; 4 UC Irvine Charlie Dunlop School of Biological Sciences, University of California, Irvine, CA 92697, USA; 5 Department of Research, University of California, Irvine, CA 92697, USA; 6 Department of Neurology, Division of Neuro-Oncology, University of California, Irvine, CA 92697, USA

**Keywords:** vorasidenib, anaplastic oligodendroglioma, isocitrate dehydrogenase, IDH inhibitor, glioma

## Abstract

**Background/Objectives**: Anaplastic oligodendrogliomas (AOs) are central nervous system (CNS) World Health Organization (WHO) grade 3 gliomas characterized by isocitrate dehydrogenase (IDH) mutation (m)IDH and 1p/19q codeletion. AOs are typically treated with surgery and chemoradiation. However, chemoradiation can cause detrimental late neurocognitive morbidities and an accelerated disease course. The recently regulatory-approved vorasidenib, a brain-penetrating oral inhibitor of IDH1/2, has altered the treatment paradigm for recurrent/residual non-enhancing surgically resected CNS WHO grade 2 mIDH gliomas. Though vorasidenib can delay the time to chemoradiation for grade 2 gliomas, the implications for vorasidenib in non-grade 2 mIDH gliomas are not well understood. **Results:** We present a case of a 71-year-old male with a grade 3 non-enhancing oligodendroglioma successfully treated with vorasidenib with an 11% reduction in residual tumor volume. Vorasidenib was well tolerated in our patient with a mild elevation in his liver transaminases that resolved following a brief interruption in treatment. **Conclusions:** Our case suggests that vorasidenib may impart therapeutic benefits in this setting. This case illustrates the need for further investigation into these less commonly addressed scenarios and treatment strategies that extend beyond current guidelines.

## 1. Introduction

Anaplastic oligodendrogliomas (AOs) are central nervous system (CNS) World Health Organization (WHO) grade 3 gliomas characterized by isocitrate dehydrogenase mutations (mIDHs) and 1p/19q codeletion [1,2]. The diagnostic criteria for oligodendrogliomas have undergone multiple revisions with the integration of molecular criteria to improve upon diagnostic accuracy. However, the grading criteria in the current classification schema for oligodendrogliomas continues to predominantly rely upon histopathologic examination; the differentiating criterion between grade 2 and grade 3 oligodendrogliomas are the presence of conspicuous microvascular proliferation and/or 6 mitoses per 10 high-power field [3,4,5,6,7,8,9,10,11]. These criteria are subject to inter-rater variability, and accordingly, the reliability of these benchmarks as a predictor of patient outcome have been called into question [7,12,13,14]. To ameliorate this variability, molecular alterations, including homozygous loss of cyclin-dependent kinase inhibitor (CDKN)2A and mutations in phosphatase and tensin homolog (PTEN) and NOTCH1 have been investigated as adjunctive markers for risk stratification. Nevertheless, their prognostic role and clinical relevance are yet to be determined [15,16,17].

The distinction between grade 2 and grade 3 oligodendrogliomas carries therapeutic relevance. While maximal safe resection remains the initial treatment for oligodendrogliomas, the implications for adjuvant chemotherapy and radiation are reliant upon risk stratification [18,19,20,21,22]. To date, consensus has not been reached on the risk stratification criteria for oligodendrogliomas (Table 1). Various risk factors have been identified across multiple trials, including the European Organization for Research and Treatment of Cancer (EORTC) 228445, 228446, and Radiation Therapy Oncology Group (RTOG) 0424 trials. RTOG 9802 and other studies further emphasize the impact of tumor extent of resection (EOR) as a prognostic factor [23,24,25,26,27,28]. Consequently, there is a growing focus on residual tumor volume (RTV) as a principal prognostic factor [10,27,29,30].

## 2. Detailed Case Description

**Figure 1:**
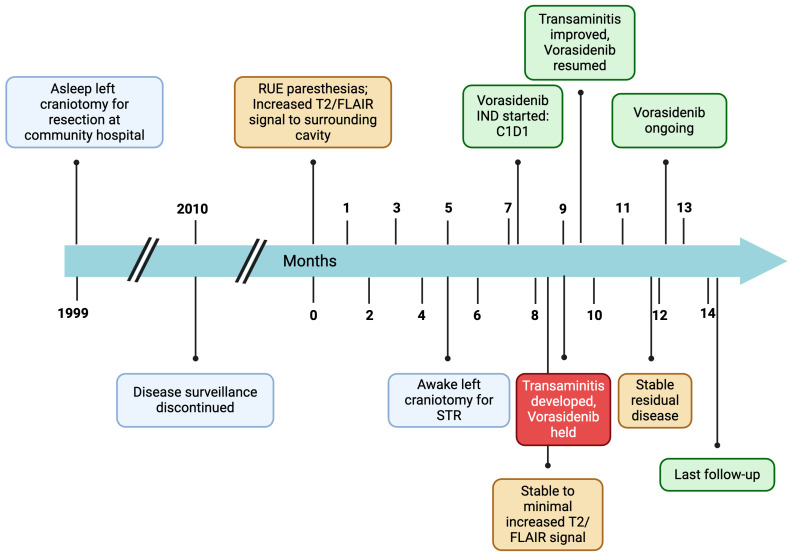
Patient disease course timeline. RUE = right upper extremity; STR = subtotal resection; IND = investigational new drug; FLAIR = fluid attenuated inversion recovery.

**Figure 2:**
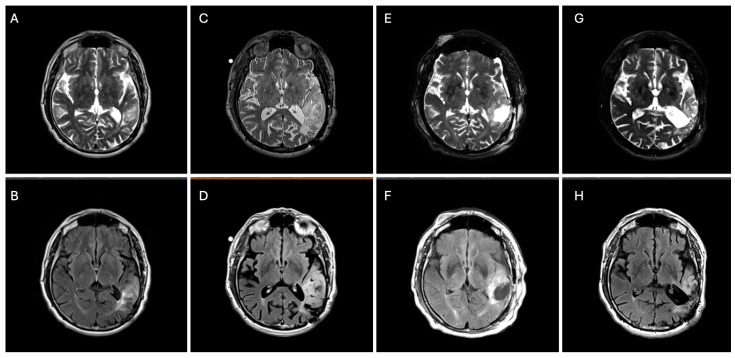
Axial T2 (top panels) and FLAIR (bottom panels) brain MRIs demonstrating an interval increase in left parietal–temporal T2/FLAIR-hyperintensity from 2017 (**A**,**B**) to 2024 (**C**,**D**) with subsequent surgical debulking (residual tumor volume 45 cm^3^) (**E**,**F**) followed by 8 months of vorasidenib (residual tumor volume 40 cm^3^) (**G**,**H**).

Twenty-five years prior to presentation, a 71-year-old ambidextrous male presented to an outside hospital with syncope (Figure 1). He was incidentally found to have a left parietal non-enhancing mass. He underwent resection at a community hospital with a diagnosis of grade 2 oligodendroglioma. The EOR is unknown. He was monitored for 11 years with no evidence of recurrence (Figure 2A,B), and surveillance was discontinued.

**Figure 3:**
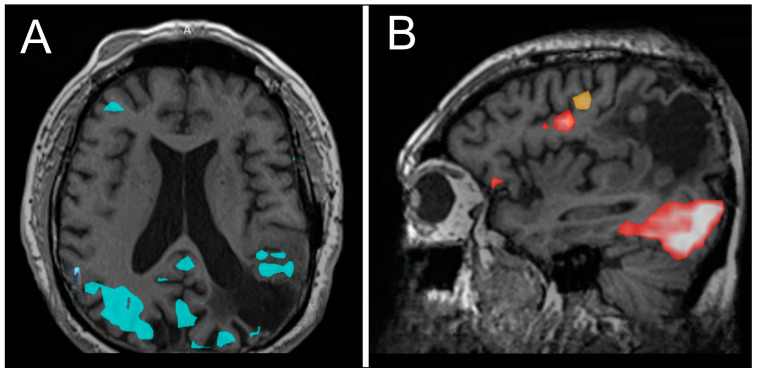
Functional MRI (fMRI) demonstrating areas of activation during lip movement (**A**), word production, and verb comprehension (**B**).

At year 24, he presented to our institution for evaluation of right arm numbness. His neurological exam showed decreased upper-right extremity sensation to light touch. Magnetic resonance imaging (MRI) of the brain showed an interval increase in T2/FLAIR hyperintensity surrounding the resection cavity with no correlative enhancement (Figure 2C–F). A functional MRI showed left-sided language lateralization at Broca’s area (Figure 3). During lip movement, there was overlap in activation of the left primary motor cortex with T2/FLAIR signal hyperintensity, but foot and hand movements were distant from the lesion. His clinical and radiographic progression warranted re-resection and tissue diagnosis to guide adjuvant therapy. A subtotal resection was achieved with approximately 2 cm of residual disease and an RTV of 45 cm^3^ remaining in eloquent structures (arcuate fasciculus, superior longitudinal fasciculus).

**Figure 4:**
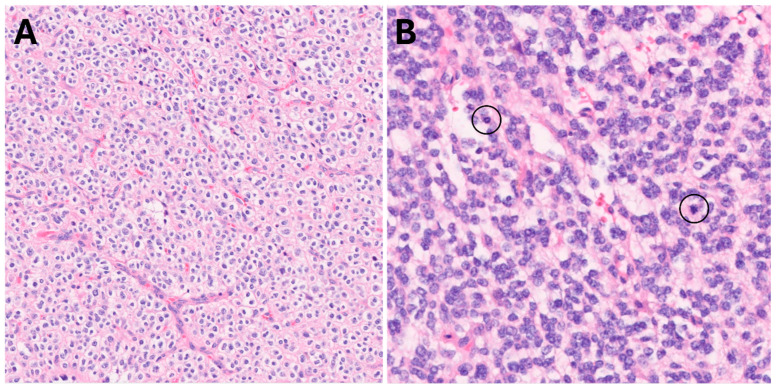
Classic oligodendroglial morphology, 200× (**A**). High-grade areas (**B**) showed brisk mitotic activity (circles) and hypercellularity, 400×.

Pathology (reviewed independently by two board-certified, fellowship-trained neuropathologists) showed a widely infiltrative glioma with classic oligodendroglial morphology, including round to oval nuclei, perinuclear halos, and thin intervening capillaries (Figure 4A). High-grade features were also present (Figure 4B), characterized by markedly cellular areas with brisk mitotic activity (up to 10 mitoses in 10 high power fields). No necrosis or microvascular proliferation was seen. Immunohistochemistry demonstrated positivity for IDH1 R132H mutant protein and ATRX expression was retained in tumor nuclei. 1p/19q co-deletion was detected by fluorescence in situ hybridization (FISH) and Tempus xT 648 gene panel reported genomic variants in IDH1, TERT, CIC, and FUBP1, as expected for an oligodendroglioma. These findings yielded a final diagnosis of oligodendroglioma, IDH-mutant and 1p/19q-codeleted, CNS WHO grade 3.

The patient’s postoperative course was uncomplicated. His mild right upper extremity weakness, paresthesia, and incoordination improved. Given his grade 3 tumor designation, chemoradiation versus vorasidenib was discussed. A consensus was reached at our multidisciplinary Neuro-Oncology Tumor Board to recommend vorasidenib. The patient decided to proceed with 40 mg of vorasidenib. Vorasidenib was well-tolerated, with the exception of asymptomatic transaminitis, which subsequently resolved spontaneously after a two-week treatment interruption. At 8-month follow-up, he remains clinically stable with radiographic findings of an 11% reduction in RTV to 40 cm^3^ (Figure 2G,H).

## 3. Discussion

The management of oligodendrogliomas following surgery is influenced by several key factors, including age, tumor grade, and EOR. Together, these components stratify the risk of recurrence (Table 1). The median age of diagnosis for oligodendrogliomas is 49.5 years of age [17], which corroborates with our patient’s age of initial diagnosis at 47 years. The role of age as a prognostic indicator for oligodendrogliomas remains a matter of debate with a historical threshold of  ≥ 40 years as the benchmark for high-risk classification in grade 2 gliomas [10]. Recent studies suggest that an older age cutoff (45, >50, or ≥60) is a more reliable indicator for identifying high-risk LGG patients [8,16,31]. Based on RTOG 9802 and EORTC 22033–26033 criteria, our patient is categorized as high-risk on the basis of his age at diagnosis. On the contrary, he is stratified to a low-risk classification under the EORTC 22844/5 and RTOG 0424 criteria (oligodendroglioma lineage, and absence of pre-operative neurological deficit, unknown preoperative tumor diameter) [10,24,28,32,33,34,35].

Our patient defied survival expectations with a disease trajectory that continues over 24 years from diagnosis, and he remains alive at time of publication. Under the “wait-and-see” strategy, the anticipated median OS is 13.2–14.2 years with a median PFS of 3.4 years for grade 2 LGGs [10,33]. His unexpected longevity may stem from the persistent lack of enhancement of his tumor at recurrence, which may indicate a less aggressive glioma compared to an enhancing glioma [36]. Despite historical controversy regarding surgical management of LGGs, early maximal safe resection is now favored over the watchful waiting approach [27,37,38,39,40]. In our patient, there was minimal residual tumor observed on his 10-year MRI, suggesting an initial near-total or gross-total resection. Early and maximal surgery offers three related benefits of reducing malignant transformation rate, reducing recurrence rate, and improving OS [23,29,30,39,40,41]. Results from a 20-year investigation conducted by Hervey-Jumper et al. suggest that ≥75% EOR positively influences survival in these tumors [42]. Our patient’s tumor also exhibited an insidious growth pattern. Based on his available imaging, his estimated growth rate was 0.27 cm^3^ per month, or a volume of diametric expansion (VDE) of 0.87 mm per year. VDE, the rate of change in tumor diameter over time, is an independent predictor of outcome in LGGs [43,44,45]. This insidious growth rate may have contributed to the protracted quiescent period prior to his tumor’s malignant transformation. Gozé et al. showed that LGGs with a VDE of < 8 mm/year are less likely to undergo malignant transformation compared to LGGs with accelerated growth rates (36.1% vs. 73.9%, respectively) and exhibit prolonged time to malignant transformation (71.2 vs. 47.1 months, *p* < 0.001) [43]. Lastly, our patient had a low RTV following his initial surgery. The benefit of a smaller RTV is substantiated in several studies and associated with a decreased risk of recurrence and improved OS [29,30]. RTV > 10–15 cm^3^ is associated with earlier progression and decreased survival [29,30]. Following his second resection, our patient’s RTV was 45 cm^3^ with a 2 cm diameter owing to tumor in eloquent language cortex.

At the time of initial diagnosis, our patient’s oligodendroglioma diagnosis was established based on the WHO 1993 grading criteria, which were exclusively based on histomorphology. Though molecular analyses were not obtained for our patient’s initial tumor, molecular analyses obtained from his re-resection confirmed that his tumor harbors the canonical IDH1-R132H and codeletion of 1p/19q. Further, our patient’s tumor underwent malignant degeneration to grade 3 with findings of brisk mitotic activity, though microvascular proliferation and necrosis were absent and thus fulfilling only one criterion for a grade 3 designation [14]. Our patient’s tumor harbored mutations in TERT, FUBP1, and CIC, which are common alterations in oligodendroglioma, but not clinically relevant [46,47]. A rare finding in oligodendrogliomas, CDKN2A/B is the sole molecular marker to distinguish grade 2 from grade 3 oligodendrogliomas [17] but was not present in our patient’s tumor. Accordingly, our patient’s tumor met the criteria for a grade 3 designation based on a solitary histologic feature alone.

Our patient would have been ineligible for the INDIGO trial and is ineligible for the regulatory-approved drug based on his tumor’s grade 3 designation. At the time of recurrence, our patient was elderly, his tumor malignantly transformed, and he had residual tumor. Taken together, adjuvant therapy was warranted. In view of the well-documented grade 3 and 4 hematologic toxicities associated with chemoradiation, we reasoned that vorasidenib was the optimal therapy for our patient due to its low toxicity profile, his non-enhancing residual disease, and a grade 3 determination based on brisk mitotic activity alone in the absence of microvascular proliferation, necrosis, and intact CDKN2A/B. His tumor is conceivably an intermediate-risk oligodendroglioma, a proposed new category of grade 3 oligodendrogliomas [48]. On these bases, vorasidenib was favored over sequential chemoradiation with the goal of halting disease progression and prolonging the time to his next intervention. Arguably, the majority of cases in the INDIGO trial were high-risk gliomas warranting chemoradiation [48]. However, despite this high-risk assignment, PFS was improved in the vorasidenib group [49].

At last follow-up, our patient was clinically stable with radiographic improvement of an 11% reduction in RTV (40 cm^3^). Although a defined RTV cutoff for benefit with maintenance vorasidenib has yet to be determined, our case suggests that vorasidenib has a beneficial effect despite a substantial RTV. He continues to tolerate treatment well. His transient grade 1 liver transaminase elevation resolved with a brief interruption in treatment, which can be observed in 9.6% of patients who develop ≥ grade 3 elevated alanine aminotransferase [49]. Limited evidence suggests that IDHi responders lack NOTCH1 mutations [50]. Though our patient’s tumor lacks NOTCH1 mutations, it is unclear if this is linked to his response to vorasidenib. Vorasidenib is FDA indicated for use in grade 2 oligodendroglioma but may have greater potential, including in non-enhancing grade 3 oligodendrogliomas as substantiated by our case.

We acknowledge that the limitations of this study are the nature of a case report as a single case is not generalizable to all grade 3 oligodendrogliomas. Though a 11% reduction in RTV was demonstrated in our case, we could not evaluate for long-term response given the short follow-up. Subsequent analyses from the INDIGO trial demonstrated decreased tumor growth rate as measured by percent change in volume in 6-month intervals [49]. Given our patient’s prolonged disease course, and initial diagnosis during the pre-molecular era, molecular analysis was not performed for the patient’s initial tumor tissue. Moreover, DNA methylation profiling was not performed on his tumor tissue, which may have been informative to identify the favorable glioma-CpG island (G-CIMP)-high methylated phenotype from a less favorable G-CIMP-low phenotype [51,52,53]. Lastly, we could not confirm the initial RTV nor determine his initial preoperative VDE given that he was under active surveillance at an outside institution with irregular monitoring.

Future investigations should be directed at recognition of additional cohorts of LGG patients who may benefit from vorasidenib and identifying the utility of IDHis in non-grade 2 and enhancing gliomas. Accordingly, investigations with safusidenib (DS-1001) and olutasidenib, both of which are oral brain-penetrant IDH inhibitors with activity in enhancing gliomas, are underway. Safusidenib is a selective IDH R132 inhibitor that has explicitly demonstrated benefit in an enhancing grade 3 oligodendroglioma [54]. This is a notable finding, given that there are differences in outcome with vorasidenib in canonical mIDH1 R132H gliomas compared to non-canonical mIDH gliomas [55]. In patients treated with vorasidenib, it is unknown if IDH inhibition and alteration of this pathway will modify the tumor’s biology and if this inhibition will confer resistance to future chemoradiation [48]. Conversely, another open area of investigation is the role of maintenance vorasidenib in patients previously treated with chemoradiation. Forthcoming studies should also be directed at identifying epigenetic changes within additional affected signaling pathways, such as RB and PI3K [56]. These studies may detect actionable targets for use in combinatorial therapy with vorasidenib [56,57]. As our understanding of IDH-mutant gliomas tumorigenesis evolve, therapies will also continue to advance.

## 4. Conclusions

The grading criteria for oligodendrogliomas is predominantly based on subjective histopathologic features. Oligodendrogliomas may be classified as grade 3 based on brisk mitotic activity alone. We present the successful use of vorasidenib in a novel clinical context of an individual with a recurrent malignantly transformed chemoradiation-naïve oligodendroglioma with an acceptable toxicity profile. Vorasidenib may have indications that extend beyond current guidelines.

## Figures and Tables

**Table 1:** Variable criteria for risk stratification for oligodendrogliomas.

Study Title	Age	Lineage	Tumor Size	Tumor Location	Neurological Deficit	Risk
EORTC 22844/5	Age ≥ 40 years	Astrocytic	≥6 cm or crossing midline	NA	Preoperative deficit	Low risk: <3 Risk factors High risk: ≥3 Risk factors
EORTC 22033-26033	Age ≥ 40 years	Progressive disease	≥5 cm	Tumor crossing midline	Preoperative deficit	Low risk: <1 Risk Factor High risk: ≥1 Risk factor
RTOG 0424	Age ≥ 40 years	Astrocytic	≥6 cm	Tumor crossing midline	Preoperative deficit	Low risk: <3 Risk factors High risk: ≥3 Risk factors
RTOG 9802	Age ≥ 40 years OR Age ≥ 18 years with subtotal resection					Low risk: <1 Risk Factor High risk: ≥1 Risk factor

## Data Availability

The data presented in this study are available on request from the corresponding author due to privacy and ethical restrictions.

## Abbreviations

2-HG	2-hydroxyglutarate
AO	anaplastic oligodendroglioma
CDKN	cyclin-dependent kinase inhibitor
CNS	central nervous system
EOR	extent of resection
EORTC	European Organization for Research and Treatment of Cancer
FLAIR	fluid attenuated inversion recovery
fMRI	functional MRI
G-CIMP	glioma-CpG island
IDH	isocitrate dehydrogenase
IDHis	isocitrate dehydrogenase inhibitors
IND	investigational new drug
LGG	low-grade glioma
mIDH	isocitrate dehydrogenase mutations
MRI	magnetic resonance imaging
OS	overall survival
PCV	procarbazine, CCNU, and vincristine
PFS	progression-free survival
PTEN	phosphatase and tensin homolog
RTOG	Radiation Therapy Oncology Group
RTV	residual tumor volume
RUE	right upper extremity
STR	subtotal resection
TMZ	temozolomide
VDE	volume of diametric expansion
WHO	World Health Organization
